# Artificial Intelligence Applications in Gastric Cancer Surgery: Bridging Early Diagnosis and Responsible Precision Medicine

**DOI:** 10.3390/jcm15062208

**Published:** 2026-03-13

**Authors:** Silvia Malerba, Miljana Vladimirov, Aman Goyal, Audrius Dulskas, Augustinas Baušys, Tomasz Cwalinski, Sergii Girnyi, Jaroslaw Skokowski, Ruslan Duka, Robert Molchanov, Bojan Jovanovic, Francesco Antonio Ciarleglio, Alberto Brolese, Kebebe Bekele Gonfa, Abdi Tesemma Demmo, Zilvinas Dambrauskas, Adolfo Pérez Bonet, Mario Testini, Francesco Paolo Prete, Valentin Calu, Natale Calomino, Vikas Jain, Aleksandar Karamarkovic, Karol Polom, Adel Abou-Mrad, Rodolfo J. Oviedo, Yogesh Vashist, Luigi Marano

**Affiliations:** 1 Department of General Surgery and Surgical Oncology, “Saint Wojciech” Hospital, “Nicolaus Copernicus” Health Center, 80-000 Gdańsk, Poland; s.malerba10@studenti.uniba.it (S.M.); tcwalinski@copernicus.gda.pl (T.C.); sgirnyi@copernicus.gda.pl (S.G.); jskokowski@copernicus.gda.pl (J.S.); 2 Department of Precision and Regenerative Medicine and Ionian Area, University of Bari “Aldo Moro”, 70110 Bari, Italy; mario.testini@uniba.it (M.T.); francesco.prete@uniba.it (F.P.P.); 3 Department of Surgery, University Medical Center OWL—Campus Hospital Lippe, 32756 Detmold, Germany; miljana.vladimirov@gmail.com; 4 Department of General Surgery, Mahatma Gandhi Medical College and Research Institute, Pondicherry, Cuddalore Rd, ECR, Pillayarkuppam, Puducherry 607402, India; doc.aman.goyal@gmail.com; 5 Department of Medicine, Adesh Institute of Medical Sciences and Research, Bathinda 151001, Punjab, India; 6 Institute of Clinical Medicine, Faculty of Medicine, Vilnius University, 03101 Vilnius, Lithuania; audrius.dulskas@gmail.com; 7 Department of Surgical Oncology, National Cancer Institute, 03101 Vilnius, Lithuania; 8 Institute of Biosciences, Life Science Center, Vilnius University, 01513 Vilnius, Lithuania; augustinas.bausys@mf.vu.lt; 9 Department of General and Abdominal Surgery and Oncology, National Cancer Institute, 08406 Vilnius, Lithuania; 10 Department of Medicine, Academy of Applied Medical and Social Sciences-AMiSNS (Akademia Medycznych I Spolecznych Nauk Stosowanych), 52-300 Elbląg, Poland; k.polom@amisns.edu.pl (K.P.); yogeshvashist@gmail.com (Y.V.); 11 Department of Surgery, Dnipro State Medical University, Volodymyra Vernadskoho St. 9, 49044 Dnipro, Ukraine; rusduka@gmail.com (R.D.); rob_molch@yahoo.com (R.M.); 12 Department of Anesthesiology, Emergency Center, University Clinical Center of Nis, 18000 Nis, Serbia; bokistet@gmail.com; 13 Faculty of Medicine, University of Belgrade, 11000 Belgrade, Serbia; 14 Department of General Surgery and HPB, Azienda Ospedaliera Perugia, 06129 Perugia, Italy; francesco.ciarleglio@ospedale.perugia.it; 15 Department of General Surgery and HPB, Azienda Provinciale per i Servizi Sanitari di Trento (APSS), 38123 Trento, Italy; alberto.brolese@apss.tn.it; 16 Department of Surgery, Madda Walabu University, Goba 4540, Ethiopia; kebebeb21@gmail.com (K.B.G.); demmoabdi2@gmail.com (A.T.D.); 17 Department of Surgery, Medical Academy, Faculty of Medicine, Lithuanian University of Health Sciences, 50161 Kaunas, Lithuania; zilvinas.dambrauskas@lsmu.lt; 18 Department of Surgery, Hospital Regional Maria Inmaculada, Florencia 180001, Colombia; perez.adolfo21@gmail.com; 19 Department of Surgery, University of Medicine and Pharmacy Carol Davila, 010001 Bucharest, Romania; valentin.calu@umfcd.ro; 20 Kidney Transplant Unit, Department of Medicine Surgery and Neuroscience, University Hospital of Siena, 53100 Siena, Italy; natale.calomino@unisi.it; 21 Department of Surgical Oncology, HCG Manavata Cancer Centre, Nashik 422001, India; drvickyjain@gmail.com; 22 Zvezdara University Clinical Center, “Nikola Spasić” Surgical Clinic, Faculty of Medicine, University of Belgrade, Dimitrija Tucovica 161, 11000 Belgrade, Serbia; alekara@sbb.rs; 23 Faculty of Medicine, University of Belgrade, Dr Subotica 1, 11000 Belgrade, Serbia; 24 Department of Surgery, Centre Hospitalier Universitaire d’Orléans, 45000 Orléans, France; adel.abou-mrad@orange.fr; 25 Department of Surgery, Nacogdoches Medical Center, Nacogdoches, TX 75962, USA; roviedo3@central.uh.edu; 26 Department of Surgery, Tilman J. Fertitta Family College of Medicine, University of Houston, Houston, TX 77001, USA; 27 Department of Surgical Oncology, General Visceral Thoracic and Vascular Surgery, Dietrich-Bonhoeffer-Clinic, 17033 Neubrandenburg, Germany

**Keywords:** artificial intelligence, augmented reality, computer vision, gastric cancer, machine learning, predictive modeling, robotic surgery, surgical ethics

## Abstract

**Background**: Artificial intelligence is emerging as a promising tool in surgical oncology, with growing evidence suggesting potential applications in diagnostic support, intraoperative guidance, and perioperative risk assessment. In gastric cancer surgery, emerging applications range from AI-assisted endoscopic detection to data-driven perioperative risk prediction, while some technological developments, particularly in robotic autonomy, derive from broader surgical or experimental models that may inform future gastric procedures. **Methods**: A narrative review was conducted following established methodological standards, including the Scale for the Assessment of Narrative Review Articles (SANRA) and the Search–Appraisal–Synthesis–Analysis (SALSA) framework. English-language studies indexed in PubMed, Scopus, Embase, and Web of Science up to October 2025 were included. Evidence was synthesized thematically across five domains: AI-assisted anatomical recognition and lymphadenectomy support, autonomous robotic systems, early cancer detection, perioperative predictive and frailty models, and ethical and regulatory considerations. **Results**: AI-based computer vision and deep learning algorithms have demonstrated promising capabilities for real-time anatomical recognition, surgical phase classification, and intraoperative guidance, although evidence of direct patient-level benefit remains limited. In diagnostic settings, AI-assisted endoscopy and Raman spectroscopy have been shown to improve early lesion detection and reduce dependence on operator experience. Predictive models, including MySurgeryRisk and AI-driven frailty assessments, may support individualized prehabilitation planning and perioperative risk stratification. Persistent limitations include small and heterogeneous datasets, insufficient external validation, and unresolved concerns related to data privacy, algorithmic interpretability, and medico-legal responsibility. **Conclusions**: Artificial intelligence is progressively emerging as a promising tool in gastric cancer surgery, integrating automation, advanced analytics, and human clinical reasoning. Its safe and ethical adoption requires robust validation, transparent governance, and continuous surgeon oversight. When developed within human-centered and ethically grounded frameworks, AI can augment, rather than replace, surgical expertise, potentially advancing precision, safety, and equity in oncologic care.

## 1. Introduction

Artificial Intelligence (AI), first conceptualized during the Dartmouth Conference in 1956, has progressively evolved into a transformative computational paradigm influencing multiple scientific and technological domains. The United States National Artificial Intelligence Act (2020) defines AI as machine-based systems capable of generating predictions or decisions that affect real or virtual environments under human-defined objectives [1]. With the development of Deep Learning (DL) and Deep Neural Networks (DNNs), computational models have achieved remarkable capacity to identify complex, non-linear patterns within large heterogeneous datasets [2]. These advances have enabled performance levels comparable to, and in some tasks exceeding, human capabilities in fields such as image recognition and speech processing, positioning AI as a key component of contemporary computational medicine [3].

Within surgical practice, AI has been increasingly investigated as a tool to augment technical precision and clinical decision-making. At the same time, important challenges remain, including automation bias, data imbalance, and limited interpretability of algorithmic outputs, as highlighted by Hashimoto et al. [4].

Gastric cancer continues to represent a major global health burden, ranking fifth in incidence and fourth in cancer-related mortality worldwide, with approximately 969,000 new cases and 660,000 deaths reported in 2022 [5]. Over recent decades, minimally invasive and robotic techniques have significantly transformed its surgical management. Robotic platforms, introduced in the early 1990s, incorporated innovations such as tremor filtration, motion scaling, articulated instruments with seven degrees of freedom, and three-dimensional visualization [6]. These technological advances have facilitated smaller incisions, reduced blood loss, and faster postoperative recovery compared with traditional open surgery [7,8].

More recently, the convergence of AI with robotic surgery has begun to introduce elements of adaptive intelligence into the operative environment. Reinforcement-learning algorithms allow systems to refine performance through continuous analysis of procedural data, enabling real-time anatomical recognition, identification of safe dissection planes, and context-aware intraoperative decision support [9]. In gastric cancer surgery, such technologies may contribute to safer lymphadenectomy and improved operative consistency, particularly for surgeons with limited experience.

For instance, Kinoshita et al. demonstrated an AI-assisted laparoscopic platform capable of recognizing key gastric anatomical landmarks and vascular structures in real time, providing support for both expert surgeons and trainees [10]. Nevertheless, the reliability and generalizability of these DNN-based systems remain strongly dependent on the availability of large, well-annotated datasets and rigorous external validation [11].

The conceptual foundation of predictive computational medicine predates contemporary AI applications. Earlier algorithmic scoring systems, including APACHE for intensive care mortality prediction [12], CHA_2_DS_2_-VASc for thromboembolic risk [13], and the Multidimensional Prognostic Index (MPI) for frailty assessment [14,15,16], illustrated the clinical value of structured data-driven risk stratification. Current research is extending these principles through machine-learning models capable of integrating complex clinical variables and dynamically supporting intraoperative and perioperative decision-making [17].

Modern surgical environments generate vast volumes of multimodal data, including medical imaging, genomic information, histopathology data, physiological monitoring data, and high-definition surgical videos. When considered independently, these data streams often provide fragmented insights. AI-based analytical frameworks offer the possibility of integrating such heterogeneous information into interpretable predictive models capable of anticipating complications, improving surgical planning, and supporting personalized patient management [18].

**Figure 1:**
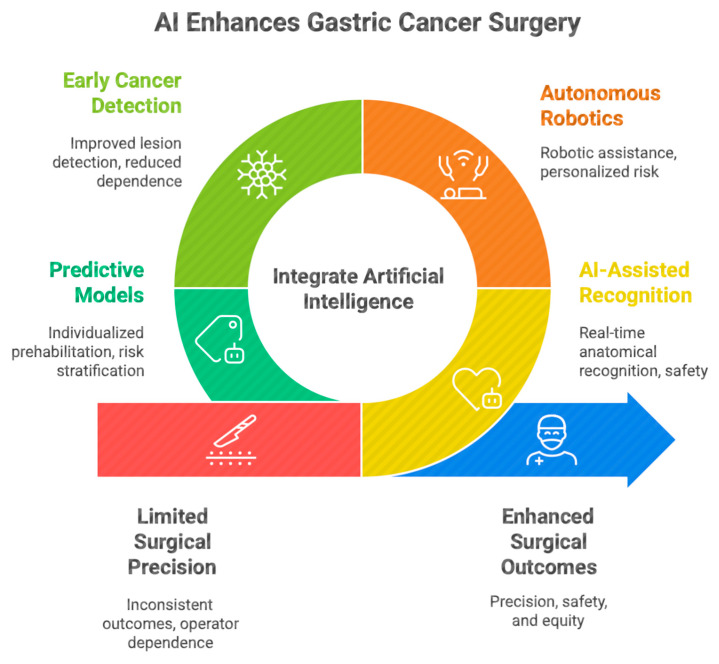
Conceptual framework illustrating the integration of artificial intelligence across the clinical continuum of gastric cancer surgery, from early endoscopic detection and diagnostic support to intraoperative guidance, robotic assistance, and perioperative risk prediction. The diagram highlights how data-driven technologies interact with surgical expertise to support precision decision-making throughout the patient pathway.

In gastric cancer surgery specifically, computer vision algorithms applied to laparoscopic and robotic video recordings have demonstrated promising applications in surgical-phase recognition, anatomical mapping, and objective performance assessment. In parallel, data-driven simulation platforms are emerging as powerful tools for accelerating technical skill acquisition and standardizing surgical training. This narrative review examines the current and emerging roles of artificial intelligence in gastric cancer surgery across technical, educational, and ethical dimensions, with particular attention to intraoperative guidance, surgical training, and perioperative risk prediction (Figure 1). As emphasized by Lim et al., the responsible integration of AI into clinical practice requires transparent validation processes, continuous human oversight, and clearly defined medico-legal accountability frameworks [19]. Artificial intelligence therefore has the potential to reshape the entire surgical pathway—from early cancer detection to intraoperative support and postoperative risk stratification—by embedding data-driven insights into clinical decision-making. Through improved anatomical recognition, enhanced robotic assistance, and predictive perioperative modeling, AI technologies may contribute to reducing variability in surgical decision-making and supporting more standardized approaches, although further clinical validation is required.

## 2. Materials and Methods

This review followed established guidance for narrative synthesis [20] and the SALSA framework (Search, Appraisal, Synthesis, Analysis). In contrast to systematic reviews, which typically address narrowly defined questions using quantitative synthesis, this approach provides conceptual integration across heterogeneous bodies of literature [21].

### 2.1. Search Strategy

A comprehensive search was conducted in MEDLINE (via PubMed), Embase, Scopus, and Web of Science up to October 2025. Search terms combined “gastric cancer,” “stomach cancer,” “gastrectomy,” and “training” with AI-related keywords (“artificial intelligence,” “machine learning,” “deep learning,” “robotics”). Reference lists of relevant articles were also screened for additional sources. Only English-language, peer-reviewed full-text articles were included. The search process followed PRISMA transparency principles [22].

### 2.2. Selection Criteria

Inclusion criteria encompassed any clinical, technical, or educational study addressing artificial intelligence or related technologies in gastric cancer surgery. Exclusion criteria included non-gastric studies, non-AI-related publications, non-research articles, animal-only studies, and abstracts without full text. Two reviewers (S.M., A.G.) independently screened titles, abstracts, and full-text articles, resolving discrepancies by consensus.

### 2.3. Data Extraction and Narrative Appraisal

Two reviewers (M.V., Y.V.) independently extracted author, year, country, study design, AI modality, clinical context, and outcomes using standardized forms. Formal quality scoring or risk-of-bias assessment was not performed, as this review follows a narrative synthesis approach. The SANRA framework was used to guide the methodological structure and reporting transparency of the review, while the included studies were descriptively analyzed according to their methodological characteristics and relevance to the topic. Discrepancies were resolved by consensus.

### 2.4. Synthesis

Thematic synthesis grouped the literature into five domains reflecting the main areas of AI application in gastric cancer surgery:

(i) AI-assisted anatomical recognition and lymphadenectomy support;
(ii) Progression toward autonomy and surgical workflow recognition;
(iii) AI for early detection and diagnostic endoscopy;
(iv) Predictive and frailty-oriented perioperative models
(v) Ethical, regulatory, and medico-legal considerations.

Given methodological heterogeneity, meta-analysis was not attempted. Instead, findings were integrated narratively to identify conceptual trends, limitations, and research gaps.

This methodology, anchored in SALSA and SANRA frameworks, ensures transparency and interpretive depth, while reflecting the evolving maturity of AI research in gastric cancer surgery.

## 3. Results of the Search and Scope of Evidence

The search identified a heterogeneous yet expanding body of literature encompassing technical, clinical, and educational studies related to artificial intelligence in gastric cancer surgery. While several investigations are directly focused on gastric procedures, others derive from related gastrointestinal or minimally invasive surgical contexts and are included to illustrate broader technological developments relevant to gastric surgery. The evidence includes convolutional neural networks for anatomical segmentation, reinforcement learning frameworks for robotic automation, and hybrid systems integrating multimodal data for surgical guidance.

Across this literature, limitations include small sample sizes, single-center datasets, inconsistent annotation, and limited external validation, highlighting the need for multicenter, standardized, and ethically transparent development of surgical AI systems. Building on these five domains, the discussion examines how AI progressively integrates along the surgical continuum, from anatomical mapping to ethical governance, providing a cohesive framework for understanding its technical and clinical implications. It is important to note that much of the current literature on artificial intelligence in gastric cancer surgery remains exploratory. Many reported systems have been evaluated in retrospective datasets, experimental environments, or limited single-center studies with heterogeneous annotation standards. Consequently, algorithmic performance metrics—such as detection accuracy or classification performance—should not be interpreted as direct evidence of improved patient outcomes. In interpreting the available evidence, it is therefore useful to distinguish between different levels of maturity, including technical feasibility, algorithmic performance, potential clinical utility within surgical workflows, and demonstrated benefit for patient-centered outcomes.

### 3.1. AI for Improved Anatomical Recognition in Gastric Cancer Surgery

Precise identification of gastric and perigastric structures is critical for oncologic safety during D2 lymphadenectomy. Anatomical variability remains a major challenge, even for experienced surgeons, with incomplete recognition predisposing to bleeding and inadequate lymphadenectomy [23].

Chen et al. proposed a real-time perigastric blood vessel recognition model (PGBVRM) for laparoscopic gastrectomy, demonstrating that automated detection of exposed vessels facilitates accurate identification and may reduce vascular injury [24]. Similarly, Kumazu et al. developed an AI-based algorithm for automated segmentation of lymphatic connective tissue during robot-assisted gastrectomy, validated quantitatively and through expert review [25]. Together, these studies suggest that computer vision systems may function as intraoperative support tools and educational aids, particularly through annotated surgical video feedback.

Parallel innovations in augmented reality (AR) and virtual reality (VR) enable immersive exploration of complex anatomy, offering scalable and cost-effective alternatives to cadaveric training. Although tactile realism remains limited, VR-based simulations have been shown to improve visuospatial understanding and psychomotor skills [26,27,28].

Clinically, integrating AI-based anatomical recognition with indocyanine green near-infrared fluorescence (ICG-NIRF) imaging may further enhance the visualization of vascular and lymphatic structures, potentially improving the precision and safety of D2 lymphadenectomy [29,30]. However, most systems are based on single-center datasets, and multicenter validation remains essential. Standardized annotation protocols, collaborative data sharing, and rigorous evaluation of hybrid AI–fluorescence workflows represent critical next steps toward reliable clinical adoption. As summarized in Table 1, AI is being integrated into multiple stages of gastric cancer surgery, from real-time vascular mapping and automated lymphatic segmentation to immersive augmented reality (AR) and virtual reality (VR) training and surgical phase recognition systems. The convergence of AI-based image analysis with fluorescence-guided surgery further illustrates the potential for multimodal, context-aware decision support. Collectively, these developments highlight the potential of data-driven tools to complement surgical expertise and contribute to more standardized educational and training frameworks.

### 3.2. Advances in the Development of Autonomous Robotic Surgery

The ongoing evolution of autonomous robotic systems aims to support surgical precision through enhanced motion control and stability. Many of these developments have been investigated in general laparoscopic or experimental surgical models rather than specifically in gastric cancer surgery, but they provide useful insight into potential future applications in complex oncologic procedures [32].

The conceptual framework of autonomy in surgery is often described by analogy to the Society of Automotive Engineers’ six-level hierarchy, ranging from level 0 (fully manual control) to level 5 (complete autonomy). Similarly, integrating real-time, AI-analyzed visual data into robotic systems may enable contextual awareness of the operative field, allowing the system to maintain optimal dissection planes and adaptively assist the surgeon, much like driver-assistance systems guide a vehicle to remain within its lane [33].

Automated anatomical segmentation therefore constitutes a pivotal component of this framework, providing real-time decision support that complements, rather than replaces, surgical expertise. Despite the sophistication of optical technologies, intraoperative outcomes continue to depend heavily on the surgeon’s cognitive and psychomotor performance, which may vary under conditions of stress or fatigue. In this context, AI-driven segmentation may help reduce cognitive load and enhance intraoperative safety [25].

Komatsu et al. made a notable contribution by developing a deep learning model for automated surgical phase recognition during laparoscopic distal gastrectomy for gastric cancer. Their algorithm achieved an accuracy of 88.8%, demonstrating that deep neural networks can classify operative stages with high accuracy and may contribute to workflow standardization across institutions [31]. A further step toward surgical autonomy has been illustrated by the Smart Tissue Autonomous Robot (STAR), developed by Krieger and colleagues. Although evaluated in experimental models of small-bowel anastomosis rather than in gastric surgery, the system successfully performed ex vivo and in vivo procedures with technical precision comparable to that reported for experienced surgeons [34]. The STAR system executes end-to-end laparoscopic suturing, automatically controlling bite depth, suture spacing, and tension to prevent luminal narrowing and anastomotic leakage [35]. Although these achievements mark significant milestones, translating such technology to complex gastric reconstructions remains a formidable challenge that requires rigorous validation and safety evaluation.

From both ethical and professional perspectives, increasing autonomy must remain coupled with continuous human oversight, particularly in high-risk oncologic procedures that require nuanced intraoperative judgment [36,37]. Hybrid models, in which selected sub-tasks are automated under direct surgical supervision, appear to represent the most acceptable and feasible pathway toward clinical adoption.

Most recently, Kim et al. introduced the Surgical Robot Transformer–Hierarchy (SRT-H) framework, which integrates transformer-based architectures with hierarchical deep learning modules to enable robots to perform complex, long-duration surgical workflows. The system employs language-conditioned imitation learning, using annotated demonstrations that include both task-level commands (e.g., “grasp the gallbladder neck”) and corrective instructions for motion refinement and error recovery. When evaluated in eight cholecystectomy procedures, the framework achieved a 100% technical success rate, underscoring the feasibility of natural language–guided robotic autonomy [38]. Nonetheless, further research is warranted to determine its applicability to more complex reconstructive and oncologic procedures, including gastric anastomoses. As illustrated in Table 2, surgical robotics is evolving from passive assistance toward context-aware autonomy. Early implementations (levels 1–3) already demonstrate tangible benefits in intraoperative navigation and workflow recognition, whereas higher levels (4–5) remain largely experimental. Achieving safe clinical translation will require hybrid human–machine models with transparent decision logic, continuous validation, and explicit frameworks for surgeon oversight and accountability.

### 3.3. Artificial Intelligence in Early Detection and Diagnostic Endoscopy

Early gastric cancer can often be treated with organ-preserving endoscopic techniques when detected at a sufficiently early stage. Lesions measuring ≤20 mm and confined to the mucosal layer (N0, M0) are typically suitable for endoscopic submucosal dissection, while endoscopic mucosal resection remains appropriate for noninvasive lesions without submucosal penetration [39]. The success of these minimally invasive approaches depends critically on high-quality visualization and the operator’s interpretive accuracy. However, small or flat lesions may escape detection, particularly when endoscopic expertise is limited, potentially leading to delayed diagnosis or incomplete resection.

To address these diagnostic limitations, artificial intelligence-enhanced optical technologies have been introduced to support the real-time identification and characterization of early neoplastic changes. Although several AI-based endoscopic technologies have been evaluated in upper gastrointestinal neoplasia more broadly, emerging studies increasingly explore their application in early gastric cancer detection. Among the most promising innovations is Raman spectroscopy integrated with AI-based classifiers. Raman spectroscopy analyzes inelastic light scattering generated by molecular vibrations within tissues, producing unique spectral “fingerprints” that reflect biochemical composition. When incorporated into endoscopic imaging, these spectral signatures may assist in distinguishing malignant, dysplastic, and normal mucosal tissue. Soong et al. evaluated the SPECTRA IMDx™ system, which applies machine learning algorithms to classify spectra obtained during upper gastrointestinal endoscopy. The system accurately distinguished high-risk neoplastic lesions, including high-grade intraepithelial neoplasia and invasive carcinoma, from benign or low-grade counterparts, achieving high sensitivity and specificity even when used by nonexpert operators [40]. This finding underscores the potential of AI-assisted Raman systems to enhance diagnostic reliability and broaden access to advanced optical diagnostics.

Beyond spectroscopy, computer-aided detection systems based on deep learning, particularly convolutional neural networks, have demonstrated comparable diagnostic performance. These algorithms analyze high-definition white-light or narrow-band imaging frames, automatically highlighting suspicious regions and estimating the depth of invasion [41]. In prospective multicenter validation studies, AI-assisted endoscopy improved early gastric cancer detection and reduced interobserver variability without extending procedural time [42]. Similar findings were reported by Lei et al. [41], who conducted a prospective multicenter study showing that AI-based analysis of endoscopic images achieved high diagnostic accuracy for early gastric cancer, and by Klang et al., who demonstrated consistent performance across centers using deep learning for automated detection of early gastric lesions [42]. Collectively, these systems may function as cognitive aids that support endoscopic interpretation and reduce variability among operators.

From a surgical perspective, the incorporation of AI-guided optical diagnostics into preoperative assessment may enhance the delineation of tumor margins and improve patient selection for minimally invasive procedures. Looking ahead, Raman spectroscopy integrated with AI could also be adapted for intraoperative use, distinguishing viable from malignant tissue in real time and supporting parenchyma-sparing, function-preserving gastric surgery.

Taken together, these advances illustrate the potential of AI-assisted technologies to complement conventional endoscopic assessment by integrating molecular and image-based information. By combining spectral and image-based analytics, AI-enabled platforms hold promise for improving early detection, optimizing resection strategies, and ultimately preserving healthy gastric tissue (Table 3).

### 3.4. Artificial Intelligence-Based Predictive Models for Assessing Perioperative Complication Risk in Gastric Cancer Surgery

Before the widespread adoption of artificial intelligence (AI) in clinical practice, early discriminative models such as CURB-65, the Acute Physiology and Chronic Health Evaluation (APACHE) score, the CHA_2_DS_2_-VASc score, and the Multidimensional Prognostic Index were routinely calculated at the bedside to estimate disease severity and postoperative risk. While these tools provided valuable prognostic information, they relied on limited static parameters and stratified risk in a generalized, population-based manner.

The recent evolution of AI-driven analytics has introduced a new era of personalized risk prediction. By processing large volumes of biological, physiological, and clinical data, these algorithms enable the development of individualized, noninvasive risk models capable of improving diagnostic precision and guiding tailored treatment strategies [43]. This transition from traditional reductionist models to integrative, data-driven frameworks reflects a broader epistemological shift in medicine, from a structural and descriptive understanding of disease to a systemic and dynamic conception of biological function [44].

Contemporary studies indicate that machine learning algorithms can predict preoperative and perioperative complications with increasing accuracy [45]. Such models may contribute to more comprehensive frailty assessment by incorporating multidimensional clinical data, including physiological, cognitive, and biochemical parameters, beyond the scope of classical constructs such as Fried’s frailty phenotype. In this context, AI-derived frailty models may inform personalized prehabilitation programs, optimize perioperative care, and estimate outcomes in scenarios where surgery is deferred, thereby supporting shared decision-making between patients and clinicians [46,47].

Laparoscopic and robotic gastrectomy, while offering minimally invasive advantages, are associated with distinct postoperative risks, including atelectasis, pulmonary infection, pulmonary edema, pulmonary embolism, and respiratory failure, primarily related to continuous pneumoperitoneum. These complications are particularly relevant in patients with preexisting pulmonary disease. Careful preoperative optimization of respiratory function is therefore essential, and open surgery may represent a safer alternative in selected cases. Effective decision-making requires comprehensive preoperative evaluation and vigilant perioperative monitoring, particularly in patients with cardiopulmonary compromise. When indicated, surgical intervention should be postponed until organ function has been optimized through targeted rehabilitation and multidisciplinary management [48].

In a study by Hong et al., three machine learning algorithms were developed to predict postoperative complications following laparoscopic gastric cancer surgery. Predictor variables included age group, history of pulmonary disease, operative time, surgical type, Eastern Cooperative Oncology Group performance status, and the American Society of Anesthesiologists score. All three models demonstrated strong predictive accuracy for both laparoscopic distal and total gastrectomy, underscoring the feasibility of integrating algorithmic risk prediction into routine perioperative assessment. Although preliminary artificial neural network-based tools for postoperative risk evaluation have already been implemented at certain surgical centers, their clinical efficacy requires confirmation through large-scale prospective validation studies [49].

Complementary work by Bihorac et al. introduced MySurgeryRisk, an automated machine learning platform that utilizes routinely collected electronic health record data to estimate the probability of major postoperative complications and mortality with high sensitivity and specificity. Designed as part of an intelligent perioperative management system, MySurgeryRisk is being incorporated into real-time clinical workflows to facilitate automated, dynamic surgical risk assessment [50].

In the preoperative context, understanding how baseline health characteristics influence susceptibility to postoperative complications, even when certain risk factors are nonmodifiable, can support transparent discussions regarding surgical benefits and limitations. Accurate individualized risk estimation also allows clinicians to identify patients most likely to benefit from targeted preventive strategies. For example, a patient with stage II chronic kidney disease and albuminuria undergoing high-risk surgery faces an increased likelihood of postoperative acute kidney injury. Although this underlying risk cannot be eliminated, its recognition enables proactive modification of perioperative management, potentially reducing the incidence and severity of renal complications.

Collectively, these advances underscore the growing clinical relevance of AI-based predictive models as integral components of perioperative planning. By combining patient-specific data with automated learning algorithms, these systems may support preoperative counseling and risk stratification, although prospective clinical validation remains necessary to confirm their impact on patient outcomes (Table 4).

### 3.5. Ethical and Regulatory Challenges of Big Data in Surgery

Although the ethical implications of artificial intelligence (AI) extend far beyond gastric cancer, they form an indispensable component of any discussion on data-driven surgery. The clinical implementation of AI relies on the large-scale use of sensitive patient information, placing privacy, informed consent, and accountability at the center of ethical and legal debate. Surgeons, who frequently serve as both data generators and custodians, bear a dual responsibility to ensure the secure handling, compliant storage, and ethically appropriate reuse of operative data. Importantly, privacy risks may not arise solely at the point of data collection; they may also emerge years later as digital archives expand and shared repositories proliferate. Patients must therefore be granted comprehensive informed consent for data sharing and be clearly informed about their rights to access, rectification, restriction, and erasure, in accordance with contemporary data protection frameworks [51].

When AI models are trained on identifiable or insufficiently anonymized data, they may inadvertently encode and expose personal information. Sophisticated reidentification or adversarial attack techniques can compromise even deidentified datasets by cross-linking limited spatial and temporal details. To reduce these vulnerabilities, any algorithm intended for clinical deployment should undergo rigorous cybersecurity and penetration testing prior to certification. Additional safeguards, such as pseudonymization, encrypted federated learning, and differential privacy mechanisms, should be embedded in system design [52]. Collectively, these measures preserve the ethical principle of confidentiality, which remains inseparable from patient autonomy and human dignity [53].

Machine learning systems that evolve through continuous real-world updating introduce regulatory complexities absent in traditional, static medical software. Their adaptive behavior blurs the distinction between premarket validation and postmarket surveillance, demanding ongoing oversight throughout the system’s lifecycle. Regulators, healthcare institutions, and clinicians therefore share responsibility for developing dynamic monitoring frameworks that ensure safety while fostering innovation [54].

Equally important is the issue of data provenance, defined as the transparent documentation of a dataset’s origin, ownership, and licensing status. Because large training datasets often integrate materials of uncertain authorship, developers must verify intellectual property compliance and prevent the inadvertent incorporation of copyrighted or trademarked content into model training or output [55].

Within Europe, legislative reform is actively addressing these emerging challenges. The European Union Artificial Intelligence Act (2024) designates surgical robotic AI systems as high-risk technologies, mandating transparent documentation, traceability, and continuous human oversight throughout their development and deployment. Nonetheless, the question of liability remains unsettled. When partially autonomous systems contribute to adverse outcomes, the allocation of responsibility among manufacturers, operators, and healthcare providers becomes increasingly complex. The European Parliament’s 2017 Resolution on Civil Law Rules for Robotics even introduced the concept of “electronic personhood,” envisioning a legal entity status that could, in theory, assign direct liability to autonomous machines [56]. Although largely theoretical and widely debated, such proposals underscore the need to redefine accountability in an era in which surgical robots may operate with limited human intervention. In the context of surgical AI, these ethical principles translate into several practical challenges. Many contemporary AI systems are trained using large datasets of operative videos, raising important questions regarding data provenance, annotation quality, and institutional governance of surgical recordings. Ensuring that training datasets accurately represent surgical variability and maintain high annotation standards is essential for preventing algorithmic bias and maintaining clinical reliability. In addition, AI-assisted surgical systems require continuous post-deployment monitoring to detect performance drift as technologies interact with evolving clinical environments. Even when advanced decision-support systems are integrated into operative workflows, the surgeon remains ultimately responsible for interpreting algorithmic outputs and maintaining safe operative judgment.

Ultimately, the ethical integration of artificial intelligence (AI) into surgical practice demands an ethics-by-design approach, embedding privacy, transparency, and auditability within algorithmic architectures from the earliest stages of development. Surgeons must remain not only skilled operators, but also stewards of digital data and guardians of patient trust, ensuring that technological advancement continues to uphold the foundational principles of beneficence, nonmaleficence, and respect for human dignity (Table 5).

In practical surgical settings, these ethical and regulatory considerations translate into a clear professional responsibility: surgeons must remain critically engaged with the design, validation, and clinical oversight of AI tools integrated into operative care. Ensuring transparency, accountability, and patient trust will be essential for the safe and sustainable adoption of artificial intelligence in gastric cancer surgery.

## 4. Conclusions

Artificial intelligence is increasingly influencing multiple stages of the gastric cancer surgical pathway, from early diagnosis to intraoperative navigation and perioperative risk assessment, by transforming how clinical information is acquired, interpreted, and applied. AI-driven computer vision now enables real-time anatomical recognition and automated segmentation of lymphatic structures, while augmented reality and virtual reality simulation platforms are accelerating the acquisition of spatial and technical proficiency among trainees. In parallel, developments in robotic autonomy, surgical phase recognition, and image-guided assistance are contributing to greater procedural standardization and reduced interoperator variability. In the preoperative and perioperative settings, predictive algorithms such as MySurgeryRisk and AI-based frailty models have introduced dynamic, individualized approaches to risk stratification and prehabilitation planning. Importantly, several technological examples discussed in this review originate from related gastrointestinal procedures or experimental surgical models, and their direct applicability to gastric cancer surgery requires further clinical validation. Despite these promising technological developments, the clinical evidence supporting many AI applications in gastric cancer surgery remains preliminary. Most published studies rely on retrospective datasets, limited sample sizes, and heterogeneous annotation standards. As a result, improvements in algorithmic performance do not necessarily translate into measurable benefits in patient outcomes. Current AI models are constrained by small, single-institution datasets; heterogeneous annotation methods; and limited external validation. Large prospective multicenter collaborations and the establishment of open access surgical video repositories are therefore needed to ensure reproducibility, fairness, and clinical generalizability. In parallel, the next generation of AI systems must prioritize transparency, interpretability, and integration into surgical workflows in ways that support, rather than complicate, clinical decision-making. The evolving regulatory framework, exemplified by the European Union Artificial Intelligence Act (2024), further emphasizes the need for continuous oversight mechanisms capable of monitoring adaptive algorithms throughout their lifecycle. Ultimately, the future of AI in gastric cancer surgery will depend not only on technological advancement but also on the ethical and professional stewardship of surgeons. Artificial intelligence should complement, not supplant, human expertise. By preserving human oversight, safeguarding patient privacy, and embedding accountability into every stage of innovation, the surgical community can ensure that AI matures into a reliable partner in the pursuit of safer, more precise, and equitable cancer care. Future progress will depend on large multicenter datasets, standardized annotation frameworks, and prospective validation studies capable of demonstrating tangible improvements in patient-centered outcomes.

## Figures and Tables

**Table 1:** Representative applications of artificial intelligence for anatomical recognition, surgical workflow analysis, and educational support in gastric cancer surgery. The table summarizes key studies illustrating how computer vision, deep learning, and immersive simulation technologies contribute to safer lymphadenectomy, improved anatomical visualization, and standardized surgical training.

Study	Surgical Context	Study Design	Dataset/Study Size	AI Application/Technology	Main Outcome	Evidence Maturity
Chen et al., 2024 [24]	Gastric cancer surgery	Retrospective analysis of laparoscopic gastrectomy video dataset	116 laparoscopic gastrectomy videos (≈2460 extracted image frames used for training; validation on independent test videos)	Real-time perigastric blood vessel recognition model (PGBVRM)	Automated identification of perigastric vessels to assist lymphadenectomy and reduce vascular injury	Feasibility study (clinical video dataset)
Kumazu et al., 2021 [25]	Gastric cancer surgery	Deep learning model trained on robot-assisted gastrectomy surgical videos	Annotated frames extracted from robot-assisted gastrectomy videos (exact number of videos not consistently reported)	Automated segmentation of loose connective tissue and lymphatic planes	Identification of safe dissection planes during robotic D2 lymphadenectomy	Technical validation study
Neyem et al., 2024 [26]	Surgical education (general surgery/anatomy training)	Educational study evaluating VR-based anatomy training platforms	Educational cohort of medical students participating in VR-based anatomy training sessions	AI-assisted virtual reality simulation for anatomical learning	Improved visuospatial understanding and anatomy training performance	Educational feasibility
Rahimi et al., 2024 [27]	Robotic surgery training (general surgical procedures)	Systematic review of robotic surgical training methods	Not applicable (systematic review of published studies)	AI-supported simulation and performance assessment tools	Evaluation of training modalities for robotic surgery skill acquisition	Evidence synthesis (training research)
Rahmani et al., 2024 [28]	Surgical education/anatomy learning	Bibliometric and mapping study of anatomy education research	Not applicable (bibliometric analysis of scientific publications)	AI-assisted analysis of visuospatial learning and anatomy education	Identification of links between visuospatial ability and surgical/anatomical learning	Conceptual/educational research
Komatsu et al., 2024 [31]	Gastric cancer surgery	Multicenter dataset of laparoscopic distal gastrectomy videos	Multicenter surgical video dataset of laparoscopic distal gastrectomy procedures	Deep learning-based surgical phase recognition system	Automatic classification of operative phases and objective performance assessment	Multicenter technical validation
AI + ICG-NIRF imaging (multiple studies) [29,30]	Gastric cancer surgery and minimally invasive GI surgery	Clinical feasibility studies integrating fluorescence imaging with image analysis	Small clinical feasibility cohorts of patients undergoing minimally invasive gastrectomy	AI-enhanced interpretation of fluorescence-guided lymphatic and vascular visualization	Improved anatomical delineation during minimally invasive lymphadenectomy	Early clinical feasibility

**Table 2:** Evolution of autonomous functions in robotic surgery and their potential relevance to gastric cancer procedures. Several examples derive from experimental or non-gastric surgical contexts but illustrate technological developments that may inform future applications in gastric surgery.

Level of Autonomy (Analogy from Automotive Industry)	Surgical Context	Study Design	Principal Surgical Application	Representative Technology/Study	Main Outcome	Evidence Maturity
Level 1–2—Assistance	Gastric cancer surgery	Deep learning model trained on robot-assisted gastrectomy surgical videos	AI-assisted anatomical segmentation	Automated recognition of lymphatic connective tissue during robot-assisted gastrectomy (Kumazu et al., 2021 [25])	Identification of connective tissue planes supporting safer D2 lymphadenectomy	Technical feasibility (gastric surgery dataset)
Level 3—Conditional autonomy	Gastric cancer surgery	Multicenter dataset of laparoscopic distal gastrectomy videos	Surgical-phase recognition	Deep-learning phase-classification network for laparoscopic distal gastrectomy (Komatsu et al., 2024 [31])	88.8% accuracy in operative phase classification, supporting workflow analysis and objective skill assessment	Multicenter technical validation
Level 4—High autonomy	Experimental gastrointestinal surgery (small bowel)	Animal and ex vivo experimental models	Autonomous anastomosis	Smart Tissue Autonomous Robot (STAR) (Shademan et al., 2016; Saeidi et al., 2022 [32,35])	Autonomous intestinal anastomosis with consistent suture spacing and leak-resistant performance	Experimental preclinical model
Level 5—Full autonomy	Experimental general surgery model	Experimental robotic system tested in pilot surgical procedures	Complex multi-step robotic procedures	Surgical Robot Transformer–Hierarchy (SRT-H) framework (Kim et al., 2025 [38])	100% technical success in pilot robotic cholecystectomy procedures using language-conditioned imitation learning	Experimental proof-of-concept

**Table 3:** Artificial intelligence-based technologies for early detection and characterization of gastric cancer. Evidence derives from studies specifically evaluating gastric cancer or from broader upper gastrointestinal endoscopic research that may inform gastric cancer detection strategies.

Technology/Modality	Clinical Context	Study Design	Underlying Mechanism	Main Diagnostic Outcome	Evidence Maturity
Raman spectroscopy + AI (SPECTRA IMDx™) (Soong et al., 2024 [40])	Upper gastrointestinal endoscopy including gastric neoplasia	Prospective feasibility/proof-of-concept study using Raman spectral data obtained during endoscopy	Detection of biochemical and molecular alterations in tissue via inelastic light scattering; machine-learning classifier interprets spectral signatures	High sensitivity and specificity for identifying high-risk neoplastic lesions compared with histopathology	Early clinical feasibility
AI-assisted endoscopy (CNN-based CAD systems) (Lei et al., 2025; Klang et al., 2023 [41,42])	Gastric cancer detection in upper GI endoscopy	Prospective and retrospective multicenter datasets of endoscopic images and videos	Deep-learning analysis of high-definition white-light or narrow-band imaging frames for automated lesion detection and invasion-depth estimation	Diagnostic performance with AUC values often exceeding 0.90 and reduced inter-observer variability	Multicenter technical validation

**Table 4:** Predictive risk assessment approaches relevant to perioperative evaluation in gastric cancer surgery. The table distinguishes traditional clinical risk scores from machine-learning predictive models and emerging AI-based frailty or prehabilitation concepts.

Predictive Model/Study	Clinical Context	Study Design	Core Input Variables	Predicted Outcome(s)	Evidence Maturity
**Traditional clinical risk scores (non-AI)**					
APACHE, CURB-65, MPI, CHA_2_DS_2_-VASc	General medical and surgical populations	Clinical scoring systems derived from observational cohort studies	Demographics, comorbidities, physiological variables, laboratory parameters	Mortality risk, disease severity, or general morbidity	Established clinical tools
**Machine-learning predictive models**					
Hong et al., 2024 [49]	Gastric cancer surgery	Retrospective dataset of patients undergoing laparoscopic gastrectomy	Age, ASA score, ECOG performance status, operative time, surgical type, pulmonary disease	Prediction of postoperative complications following laparoscopic gastrectomy	Retrospective clinical validation
MySurgeryRisk (Bihorac et al., 2019 [50])	General surgical population	Machine-learning model using large electronic health record datasets	Multidimensional perioperative clinical variables extracted from EHRs	Prediction of major postoperative complications and mortality	Large-scale retrospective validation
**Emerging AI-driven frailty and prehabilitation models**					
AI-based frailty and prehabilitation models	Surgical oncology and geriatric surgery	Multimodal datasets integrating clinical, functional, and biological variables	Physiological indicators, functional measures, metabolic and clinical parameters	Personalized frailty assessment and perioperative risk prediction	Conceptual/early translational research

**Table 5:** Ethical and regulatory considerations in the clinical integration of artificial intelligence in gastric cancer surgery. The domains summarized derive from broader digital health governance frameworks and are applicable to surgical AI systems, including those used in gastric oncology.

Ethical/Regulatory Domain	Context of Application	Source of Evidence/Framework	Clinical Implication or Source of Risk	Recommended Mitigation or Governance Strategy	Evidence Type
Data privacy and cybersecurity	Clinical AI systems using surgical datasets and patient health records	Data protection frameworks and digital health governance literature	Re-identification of anonymized patient data; vulnerability to adversarial attacks or insecure data transfer	Differential-privacy algorithms, pseudonymization, end-to-end encryption, and mandatory cybersecurity testing before clinical deployment	Ethical and regulatory guidance
Informed consent and patient autonomy	Clinical implementation of AI-assisted decision systems	Medical ethics frameworks and data protection regulations	Insufficient disclosure regarding the role of AI in clinical decision-making; limited patient understanding of secondary data use	Tiered consent models, continuous patient education, and transparent communication regarding data use and algorithmic involvement	Ethical governance principles
Data provenance and intellectual property (IP)	Development and training of AI models using large datasets	Digital governance and intellectual property frameworks	Use of unlicensed or uncertain data sources within AI training datasets; unclear ownership of clinical data	Transparent data sourcing, dataset provenance documentation, and institutional legal review of training datasets	Regulatory compliance framework
Continuous learning and regulatory adaptation	Deployment of adaptive AI algorithms in healthcare systems	Emerging regulatory frameworks (e.g., EU Artificial Intelligence Act 2024)	Algorithmic performance may change over time beyond initial certification, creating potential oversight gaps	Dynamic regulatory monitoring, real-time algorithm auditing, and mandatory post-deployment surveillance mechanisms	Policy and regulatory framework

## Data Availability

No new data were created or analyzed in this study.
